# The impact of the implementation of bundle in the prevention of ventilator-associated pneumonia in intensive care units

**DOI:** 10.1186/1753-6561-5-S6-P70

**Published:** 2011-06-29

**Authors:** DP Cais, R Mourão

**Affiliations:** 1 Infection Control, Hospital Samaritano, São Paulo, Brazil

## Introduction / objectives

Ventilator-associated pneumonias (VAP) are the most prevalent infections in intensive care units (ICU). To reduce this rate, it is recommended the application of bundles - groups of individual practices that result in substantial improved care.

### Aim

To measure the adherence to VAP bundle, correlating with the incidence of VAP per 1000 days of mechanical ventilation (MV).

## Methods

The study was conductedin three general ICU (adult, cardiology and pediatric) of a medium sized hospital in Sao Paulo (Brazil) from June/2009 to April/2010. All patients on MV were assessed using a check list with five key measures: physiotherapy, presence of condensate in the circuit, a high head>30°, oral hygiene with chlorhexidine and manual resuscitator individual. The visits were carried out fortnightly, without notice, by the same researcher, with subsequent calculation of compliance.

## Results

At the beginning, the incidence of VAP was 20/1000 days of MV and the adherence to the measures was 15%. In the second month, the membership had increased gradually, inversely proportional to the rate of VAP. From September to December, adherence ranged from 40 to 70%, with rates of VAP from 5 to 15/1000 days of MV. In February, there was a peak (30/1000 days of MV) and good adhesion to the bundle (70%), which may reflect the increase of patient severity. Later, the Infection Control Team developed an educational work, resulting in significant decrease in VAP rate (8/1000 days of MV) and 90% adherence to the bundle.

## Conclusion

The application bundle is a feasible reality that produces good results in nosocomial infection rates. However, education and periodic training remain a fundamental process of improving health services.

## Disclosure of interest

None declared.

